# Effects of grape seed-derived proanthocyanidin B2 pretreatment on oxidative stress, endoplasmic reticulum stress and apoptosis of renal tubular epithelial cells in renal ischemia–reperfusion injury model of mice

**DOI:** 10.1007/s11255-023-03494-4

**Published:** 2023-03-19

**Authors:** Zhi-shun Wang, Bo Shu, Qi Han, Guo-hao Li, Yong-lian Guo

**Affiliations:** 1 Department of Urology, The Central Hospital of Wuhan, Tongji Medical College of Huazhong University of Science and Technology, Wuhan, People’s Republic of China; 2 Hemodialysis Center, Wuhan University of Science and Technology Hospital, Wuhan, People’s Republic of China

**Keywords:** Grape seed proanthocyanidin B2, Ischemia–reperfusion injury, Oxidative stress, Mitochondrial damage, Endoplasmic reticulum stress

## Abstract

**Purpose:**

To investigate the effect of grape seed-derived proanthocyanidin B2 (GSPB2) pretreatment on acute renal ischemia–reperfusion injury model of mice.

**Methods:**

50 mice were divided into 5 groups: Sham group: mice were treated with right nephrectomy. GSPB2 group: GSPB2 was injected intraperitoneally 45 min before right nephrectomy. IRI group: right kidney was resected and the left renal arteriovenous vessel was blocked for 45 min. GSPB2 + IRI group: GSPB2 was intraperitoneally injected 45 min before IRI established. GSPB2 + BRU + IRI group: GSPB2 and brusatol (BRU) were injected intraperitoneally 45 min before IRI established. Creatinine and urea nitrogen of mice were detected, and the kidney morphology and pathological changes of each group were detected by HE staining, PAS staining and transmission electron microscopy. Expressions of Nrf2, HO-1, GRP78, CHOP, and cleaved-caspase3 were detected by immunofluorescence staining and western blotting.

**Results:**

Morphology and mitochondrial damages of kidney in GSPB2 + IRI group were significantly alleviated than those in IRI group. Expression levels of Nrf2 and HO-1 were significantly higher in GSPB2 + IRI group than those in IRI group. Expression levels of GRP78, CHOP and cleaved-caspase3 were significantly lower in GSPB2 + IRI group than those in IRI group. However, compared to GSPB2 + IRI group, protective effects of GSPB2 pretreatment were weakened in GSPB2 + BRU + IRI group.

**Conclusions:**

GSPB2 pretreatment could alleviate oxidative stress damage and reduce apoptosis of renal tubular epithelial cells, which might be related to activating the antioxidant system, up-regulating the expression of Nrf2 and HO-1, inhibiting the expressions of GRP78, CHOP and cleaved-caspase3. However, the protective effect could be reversed by brusatol.

## Introduction

Ischemia–reperfusion injury refers to tissues or organs that restore blood flow on the basis of ischemia, and the damage is further aggravated, and even the damage is irreversible. Renal ischemia–reperfusion injury is one of the most common clinical causes of acute kidney injury. Ischemia–reperfusion injury is common in the diagnosis and treatment of clinical diseases such as kidney transplantation, cardiac surgery,

nephron-sparing surgery, shock and sepsis [1]. At present, the pathophysiological mechanism of the occurrence and development of ischemia–reperfusion injury has not been fully studied. At this stage, it is believed that the pathological process of ischemia–reperfusion injury mainly includes cell energy metabolism disorder, oxidative stress injury, calcium ion overload, microcirculation disorder, vascular endothelial cell injury, a large number of inflammatory cell infiltration, cytokine secretion, complement activation, cell necrosis and apoptosis, etc. [2, 3]. It is a complex cascade reaction process involving multiple factors and multiple systems. The current protective measures against renal ischemia–reperfusion injury include ischemic preconditioning, but there are controversies among scholars of different therapeutic effects [4, 5]. It also includes stem cell transplantation. Although stem cell transplantation has made great progress in animal experiments, there are currently few relevant clinical studies [6]. In addition, it also includes the use of new clinical drugs, such as N-acetylcysteine, AT13387, pentoxifylline, etc. [7–9]. It also includes the application of some protective gases, such as the application of argon (argon, Ar), xenon (xenon, Xe), carbon monoxide and hydrogen sulfide [10–13]. However, most intervention methods have a single target. In fact, renal ischemia–reperfusion injury involves multiple mechanisms, and each mechanism overlaps and antagonizes synergistically. At present, many plant active ingredients are natural anti-oxidant and anti-inflammatory drugs with multi-target advantages and good research value.

Proanthocyanidins are the general term for a large class of polyphenol compounds that are widely present in plants. They are widely present in grape seeds, apples, pine bark, peanuts, sorghum, cocoa beans, cherries and other plants. Among them, grape seeds are the most abundant. Among them, grape seed proanthocyanidin dimers are the most widely distributed in grape seed proanthocyanidins and have the strongest antioxidant activity [14]. Studies have shown that grape seed proanthocyanidin B2 (GSPB2) is relatively stable under gastric and duodenal digestion conditions [15], and proanthocyanidin B2 can be detected in plasma within half an hour after oral administration [16]. Therefore, grape seed proanthocyanidin B2 has better bioavailability. Previous studies have shown that proanthocyanidin B2 has strong anti-oxidative stress [17], scavenging free radicals [18], anti-arteriosclerosis [19], anti-inflammatory [20], anti-apoptosis [21], anti-tumor [22] and other biological activities. However, as far as we know, the role of grape seed proanthocyanidin B2 in renal ischemia–reperfusion injury models is rarely studied.

Therefore, this study focused on the effect of grape seed-derived proanthocyanidin B2 pretreatment on oxidative stress injury and apoptosis during acute renal ischemia–reperfusion injury in mice.

## Materials and methods

### Animals preparation and experimental design

All 50 male C57BL mice were purchased from the Experimental Animal Center of Huazhong University of Science and Technology. All experimental operations in this study were approved by the Animal Ethics Review Committee of Huazhong University of Science and Technology. Animals were housed at this Experimental Animal Research Center based on standard guidelines. All mice were maintained in a homoiothermal (20–22 °C), light-controlled (8 a.m.–8 p.m.) and air-filtered room. All animals were free to eat standard diets.

### Drugs

GSPB2 and brusatol was purchased from Aladdin reagent (Aladdin, Shanghai, China).

### Experimental protocol

All C57BL mice were anesthetized by intraperitoneal injection of phenobarbital sodium (50 mg/kg). All C57BL mice were placed flat on an electric blanket to keep their body temperature at 37 °C, followed by right nephrectomy. Then the left renal arteriovenous blood vessel was blocked with a non-damaged vascular clip for 45 min, and then the vascular clip was loosened to restore the left renal arterial and venous blood flow to create a renal ischemia–reperfusion model. After the left kidney was reperfused for 24 h, the mice in each group were sacrificed and experimental specimens were kept.

All mice were randomly divided into 5 groups: Sham group (*n* = 10): mice only underwent right nephrectomy without left kidney ischemia reperfusion; GSPB2 group (*n* = 10): mice were intraperitoneally injected with GSPB2 (30 mg/kg), and then undergo right nephrectomy 45 min later; IRI group (*n* = 10), mice underwent right nephrectomy, then left kidney ischemia for 45 min, and reperfusion for 24 h; GSPB2 + IRI group (*n* = 10), mice were intraperitoneally injected with GSPB2 (30 mg/kg) 45 min before modeling, and then underwent right nephrectomy, left kidney ischemia for 45 min, and reperfusion for 24 h; GSPB2 + BRU + IRI group (*n* = 10), mice were intraperitoneally injected with GSPB2 (30 mg/kg) and brusatol (BRU) (10 mg/kg) 45 min before modeling, and then underwent right nephrectomy, left kidney ischemia for 45 min, and reperfusion 24 h.

### Serum assays

After 24 h of reperfusion, all experimental mice were sacrificed and blood samples were collected through the inferior vena cava. After the blood samples were collected, the levels of serum creatinine and urea nitrogen were tested according to the operating instructions of the commercial test kit for serum creatinine and serum urea nitrogen (Nanjing Jiancheng Institute of Bioengineering, Nanjing, China).

### Histology

#### Hematoxylin–eosin staining

After 24 h of reperfusion, the left kidney of each group of mice was cut out. Fix the left kidney tissue with 10% neutral formalin, follow the conventional paraffin section preparation process, gradient alcohol dehydration, xylene transparency, wax immersion, paraffin embedding, section (thickness 4 μm), HE staining, light microscope observe. The extent of tubular damage to renal tissue in each group of renal tissue was assessed by an experienced pathologist using the Jablonski grade scoring method [23].

#### Periodic acid-Schiff staining

Incubate with periodic acid solution for 10 min at room temperature, wash with distilled water, incubate with Schiff reagent in a 37 °C water bath in the dark for 35 min, rinse with tap water for 10 min, stain the nucleus with hematoxylin for 2 min, rinse with tap water for 3 min, dehydrate with gradient alcohol and xylene, and mount. Observe the pathological changes of the cortex and medulla of renal tissue through a microscope.

#### Transmission electron microscope

Take a fresh 1 mm^3^ small piece of kidney tissue specimen and place it in 2.5% glutaraldehyde electron microscope fixative solution for 2 h, fix it with 1% osmium acid at 4 ℃, embed in spurr resin, slice with LKB1 ultra-thin microtome, and use lead citrate after double staining with uranyl acetate, the sections were observed with JEM-1200EX transmission electron microscope.

#### Terminal deoxynucleotidyl transferase dUTP nick end labeling (TUNEL) assay

In this experiment, a commercial in situ apoptosis detection kit was used. According to the product instructions provided by the kit manufacturer, the brief steps are as follows: after the tissue paraffin sections are rehydrated and permeated, use deionized water at a ratio of 1:100. Diluent to dilute the 2 mg/ml Proteinase K solution to a final concentration of 20 μg/ml. Add 100 μl of Proteinase K solution with a concentration of 20 μg/ml to each sample to make it completely covered, and incubate for 20 min at room temperature; rinse the sample with deionized water solution, and dilute 5 × Equilibration Buffer with deionized water at a ratio of 1:5. Add 100 μl 1 × Equilibration Buffer to each sample to completely cover the sample area to be tested, and incubate at room temperature for 15 min; Thaw Alexa Fluor 647–12-dUTP Labeling Mix on ice and prepare a sufficient amount of TdT incubation buffer. Then add TdT incubation buffer to the tissue, place the slide in a humid box, and incubate at 37 °C for 60 min. Wrap the wet box with aluminum foil to protect it from light, and then wash it with PBS 3 times for 5 min each time. DAPI was added dropwise and incubated for 5 min in the dark, the specimens were stained with nucleus, and the excess DAPI was washed away with PBST for 5 min × 4 times. Mount the slide with mounting solution containing anti-fluorescence quencher, and then observe and collect images under a fluorescence microscope. Five non-overlapping fields were randomly selected in each slice and observed, the ratio of the number of positive cells to the total number of cells in each field of view was calculated, and the average of the ratios was taken as the apoptosis index (AI).

#### Measurement of SOD, MDA and T-AOC levels in kidney

The xanthine oxidase method was used to determine the activity of SOD in the kidney tissue of each group of mice (Nanjing Jiancheng Bioengineering Institute), and the absorbance value at a wavelength of 550 nm was measured with a spectrophotometer. The content of MDA in the kidney of each group of mice was measured with thiobarbituric acid (Nanjing Jiancheng Bioengineering Institute), and the level of lipid peroxide was used to reflect the level of MDA. The spectrophotometer measures the absorbance at a wavelength of 532 nm. The total antioxidant capacity (T-AOC) (Nanjing Jiancheng Bioengineering Institute) of the kidney tissue of each group of mice was measured by colorimetry, and the absorbance value at a wavelength of 520 nm was measured with a spectrophotometer.

#### Immunofluorescence staining

The protein expressions of Nrf2, HO-1, GRP78, CHOP, and cleaved-caspase3 in the kidney tissue of each group of mice were determined by immunofluorescence staining. Specifically, the sections were deparaffinized to water, and the treated tissue sections were placed in the prepared EDTA antigen retrieval solution (0.01 M EDTA buffer, pH 9.0) for antigen retrieval. Then the sections were blocked with 10% normal goat serum at 37 °C for 30 min. Then add the primary antibody: rabbit anti-mouse Nrf-2 polyclonal antibody (Nrf2; 1:50 dilution; Wuhan Sanying Biotechnology, Wuhan, China); rabbit anti-mouse HO-1 polyclonal antibody (1: 50 dilution; Wuhan Sanying Biotechnology, Wuhan, China); rabbit anti-mouse GRP78 polyclonal antibody (GRP78; 1:100 dilution; abcam, Cambridge, UK); rabbit anti-mouse CHOP polyclonal antibody (CHOP; 1:100 dilution; abcam, Cambridge, UK); rabbit anti-mouse cleaved-caspase3 polyclonal antibody (1:50 dilution; Wuhan Sanying Biotechnology, Wuhan, China), all cultured overnight at 4 °C. Then drop the fluorescent (CY3) labeled goat anti-rabbit IgG secondary antibody (IgG, 1:100 dilution, Wuhan Sanying Biotechnology, Wuhan, China), and incubate at room temperature for 30 min. And use DAPI staining solution to stain the nucleus for 5 min. Dry the liquid on the slices with absorbent paper, mount the slides with mounting solution containing anti-fluorescence quencher, and then observe and collect the images under a fluorescence microscope. Ten fields of view (× 400) were randomly selected and each slice was analyzed by using image analysis software ImagePro-Plus 6.0. Measure the positive area (Area) and integral optical density (IOD). The mean optical density (MOD = IOD/Area) (× 100%) was used to illustrate the expression of the protein of interest.

### Western blotting

According to the previous method [24], protein was extracted and purified from mouse kidney tissue. Prepare protein samples (40 µg/lane) for gel electrophoresis, separate them on a 12% sodium dodecyl sulfate–polyacrylamide gel, and transfer to PVDF membrane (Millipore). Soak the PVDF membrane with TBST (blocking solution) containing 5% skimmed milk powder, and seal it with a shaker at room temperature for 2 h. Dilute the corresponding primary antibody with blocking solution, soak the PVDF membrane in the primary antibody incubation solution, and incubate overnight at 4 °C. TBST washes the PVDF membrane thoroughly. Dilute the corresponding HRP-labeled goat anti-rabbit secondary antibody (BA1054, 1:10,000, Wuhan Boster Biological Technology, Wuhan, China) or goat anti-mouse secondary antibody (BA-1051, 1:5000, Wuhan Boster Biological Technology, Wuhan, China), soak the PVDF membrane in the secondary antibody incubation solution, and incubate for 2 h on a shaker at room temperature. Mix the enhancement solution and the stable peroxidase solution in the ECL reagent (P1050, Beijing Pulilai Gene Technology, Beijing, China) at a ratio of 1:1, and add the working solution dropwise to PVDF. Finally, the blots were captured on light sensitive imaging film (Kodak, Rochester, NY, USA) for analysis. The antibodies used are as follows: rabbit anti-mouse Nrf-2 polyclonal antibody (Nrf2; 1:1000 dilution; Wuhan Sanying Biotechnology, Wuhan, China); rabbit anti-mouse HO-1 polyclonal antibody (1:1000 dilution; Wuhan Sanying Biotechnology, Wuhan, China); rabbit anti-mouse GRP78 polyclonal antibody (GRP; 1:1000 dilution; abcam, Cambridge, UK); rabbit anti-mouse CHOP polyclonal antibody (CHOP; 1:1000 dilution; abcam, Cambridge, UK); rabbit anti-mouse cleaved-caspase3 polyclonal antibody (1:1000 dilution; Wuhan Sanying Biotechnology, Wuhan, China).

### Statistical analysis

All data were underwent statistical analysis in SPSS version 21.0 (SPSS Inc, Chicago, IL, USA). In this study, *t* test was used to analyze the differences. A value of *P* < 0.05 was considered statistically significant.

## Results

### Grape seed proanthocyanidin B2 pretreatment could significantly improve the renal function of mice after acute renal ischemia–reperfusion injury

**Fig. 1:**
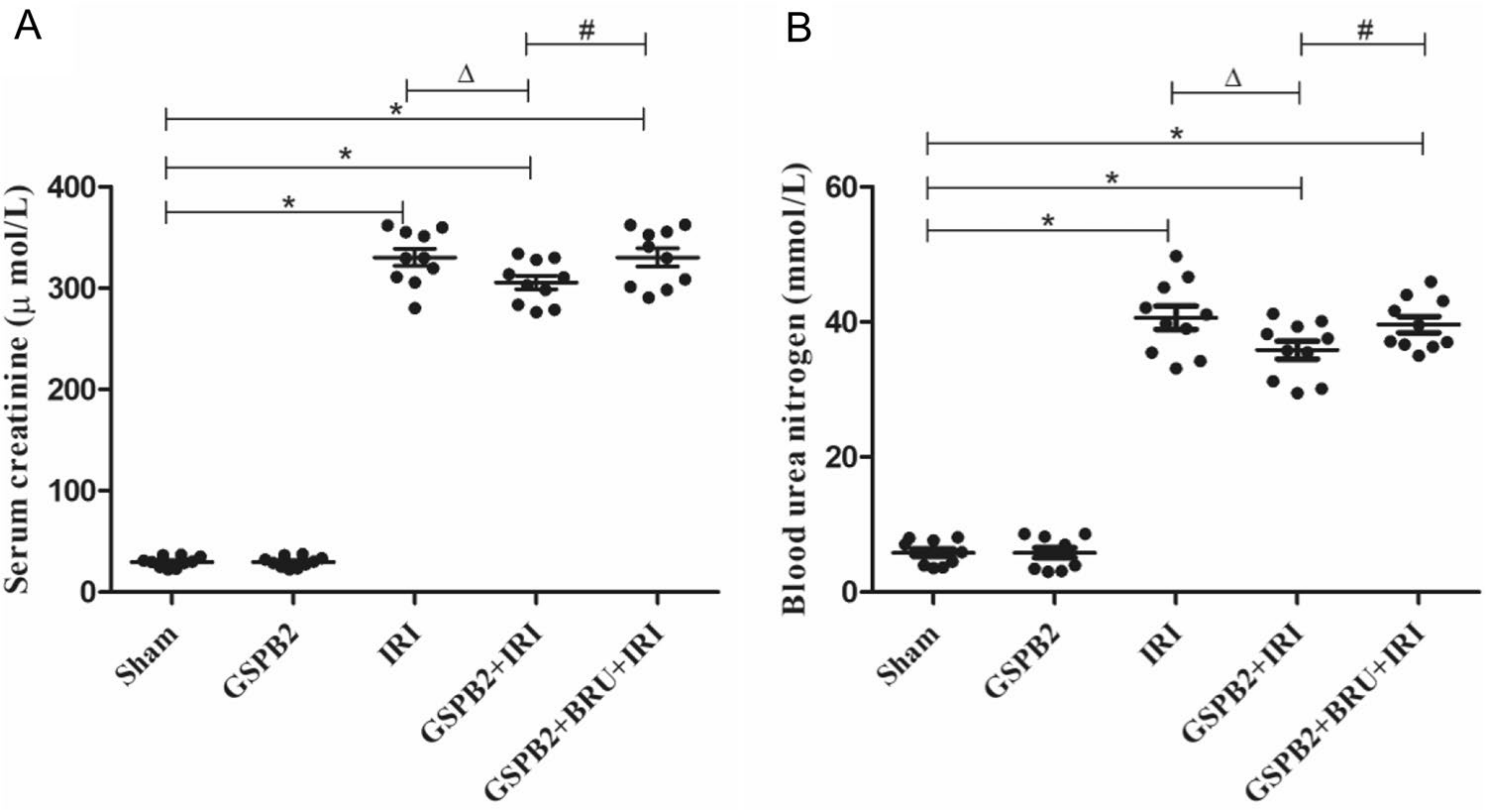
Effects of grape seed proanthocyanidin B2 pretreatment on the renal function after acute renal ischemia–reperfusion injury. **A** Serum creatinine. **B** Blood urea nitrogen. The data were presented as means ± S.D, *n* = 10 in each group. *P < 0.05 vs. group Sham. Δ*P* < 0.05 vs. group IRI. ^#^*P* < 0.05 vs. group GSPB2 + IRI

The renal function indexes of the mice in each group were detected 24 h after the renal ischemia–reperfusion injury. Compared to Sham group, the serum creatinine and urea nitrogen levels in IRI group were significantly increased, and the differences were statistically significant (*P* < 0.05). The serum creatinine and urea nitrogen levels of the GSPB2 + IRI group were significantly lower than those of the IRI group, and the differences were statistically significant (*P* < 0.05). The serum creatinine and urea nitrogen levels in GSPB2 + BRU + IRI group were significantly higher than those in GSPB2 + IRI group, and the differences were statistically significant (*P* < 0.05) (Fig. 1a, b).

### Grape seed proanthocyanidin B2 pretreatment could obviously alleviate the morphological damage after renal ischemia–reperfusion injury

**Fig. 2:**
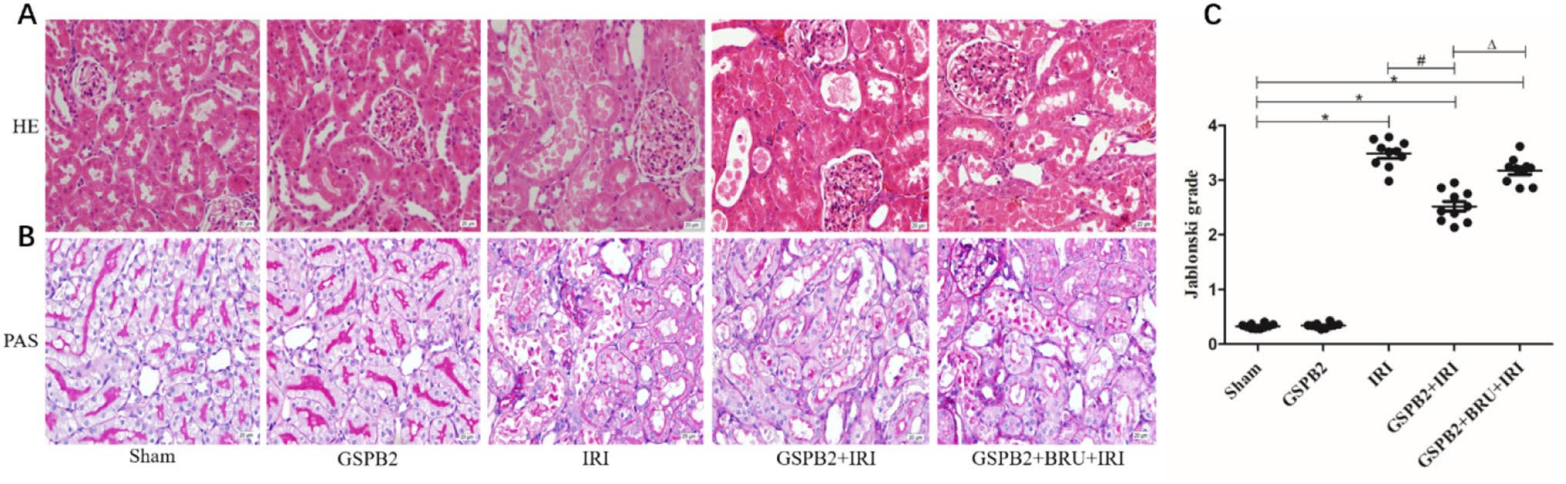
Grape seed proanthocyanidin B2 pretreatment alleviated the morphological damages in renal cortical region after renal ischemia–reperfusion according to the representative micrographs of hematoxylin–eosin staining and periodic acid-Schiff staining (× 400). **A** Hematoxylin–eosin staining. **B** Periodic acid-Schiff staining. **C** Jablonski grade. Data were shown as means ± S.D. of 10 random fields from each slide (*n* = 10 in each group). *P < 0.05 vs. group Sham. ^#^*P* < 0.05 vs. group IRI. Δ*P* < 0.05 vs. group GSPB2 + IRI

**Fig. 3:**
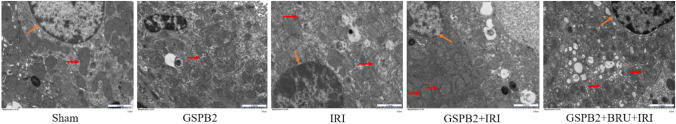
Grape seed proanthocyanidin B2 pretreatment could significantly attenuate the ultrastructural damage of renal tubular epithelial cells in renal cortical region induced by renal ischemia–reperfusion in mice (× 5 k). Transmission electron microscopy showed a series of pathological damages to renal tubular epithelial cells in the IRI group, including renal tubular cell degeneration and necrosis, mitochondrial swelling, mitochondrial vacuole degeneration, mitochondrial cristae breakage or disappearance, etc. Compared to IRI group, the ultrastructure damage of renal tubular epithelial cells in GSPB2 + IRI group was significantly reduced, especially the damage of mitochondria. However, compared to GSPB2 + IRI group, the ultrastructural damage of renal tubular epithelial cells in GSPB2 + BRU + IRI group was significantly worse. The orange arrow points to the nucleus, the red arrows point to mitochondria

HE staining, PAS staining and transmission electron microscopy were used to observe the morphological changes of mouse kidney after ischemia–reperfusion injury. HE and PAS staining showed that compared with Sham group, the swelling, necrosis and apoptosis of renal tubular epithelial cells in IRI group were significantly aggregated, some of the tubular epithelial cells were broken and lost, inflammatory cell infiltration, and kidney damage significantly worsened. Compared with IRI group, the morphological damage of the kidney in GSPB2 + IRI group was significantly alleviated. In GSPB2 + BRU + IRI group, GSPB2 and Nrf2 inhibitor brusatol were used simultaneously, and the protective effect of GSPB2 intervention was inhibited to a certain extent (Fig. 2A–C). Transmission electron microscopy showed that the kidney tissue in the IRI group had a series of damaging changes, including degeneration and necrosis of renal tubule cells, swelling of mitochondria, vacuolar degeneration of mitochondria, fracture or disappearance of mitochondrial cristae and so on. The damage of organelles in renal tubular epithelial cells in GSPB2 + IRI group was significantly attenuated than that in IRI group, especially the damage of mitochondria. In GSPB2 + BRU + IRI group, GSPB2 and Nrf2 inhibitor brusatol were used simultaneously, the protective effect of GSPB2 intervention was inhibited to a certain extent (Fig. 3).

### Grape seed proanthocyanidin B2 pretreatment could significantly reduce the apoptosis of renal tubular epithelial cells after renal ischemia–reperfusion injury

**Fig. 4:**
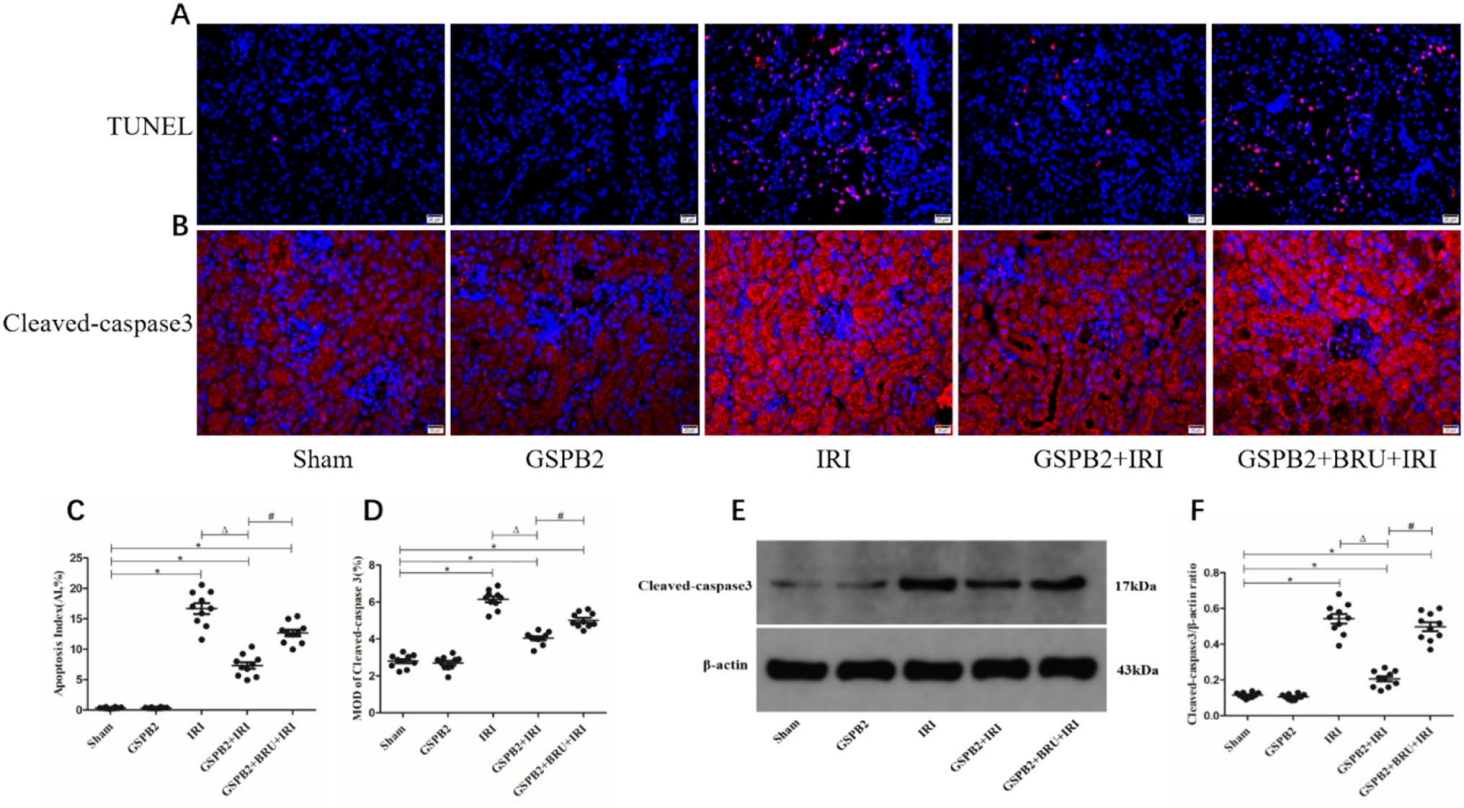
Grape seed proanthocyanidin B2 pretreatment could obviously reduce the apoptosis of renal tubular epithelial cells in renal cortical region after renal ischemia–reperfusion injury (× 400), and also could inhibit the expression level of cleaved-caspase3 protein in renal tissue of cortical region after renal ischemia–reperfusion according to the representative micrographs of immunofluorescence staining (× 400) and western blotting. **A**, **C** TUNEL staining and apoptosis index. **B**, **D** Representative micrographs of immunofluorescence staining and quantitative analysis of cleaved-caspase3. **E, F** The expression level of cleaved-caspase3 as assessed by western blotting. (*n* = 10 in each group). *P < 0.05 vs. group Sham. Δ*P* < 0.05 vs. group IRI. ^#^*P* < 0.05 vs. group GSPB2 + IRI

TUNEL was used to observe the apoptosis of renal tubular epithelial cells in each group of mice. Compared with Sham group, the number of apoptosis of renal tubular epithelial cells in IRI group and apoptosis index were significantly increased (Fig. 4A, C). At the same time, the expression level of cleaved-caspase3 protein in IRI group was significantly higher than that in Sham group (Fig. 4B, D–F). Compared with IRI group, the number of apoptosis of renal tubular epithelial cells and apoptosis index in GSPB2 + IRI group were significantly reduced, and the expression level of cleaved-caspase3 protein was significantly reduced, and the difference was statistically significant (*P* < 0.05) (Fig. 4A–F). Compared to GSPB2 + IRI group, the cleaved-caspase3 protein expression level in GSPB2 + BRU + IRI group was significantly increased, and the difference was statistically significant (*P* < 0.05) (Fig. 4B, D–F). Using GSPB2 and Nrf2 inhibitor brusatol at the same time, the protective effect of GSPB2 intervention to inhibit apoptosis was weakened to a certain extent

### Grape seed proanthocyanidin B2 pretreatment could significantly increase the activity of SOD and increase the level of T-AOC after renal ischemia–reperfusion injury

**Fig. 5:**
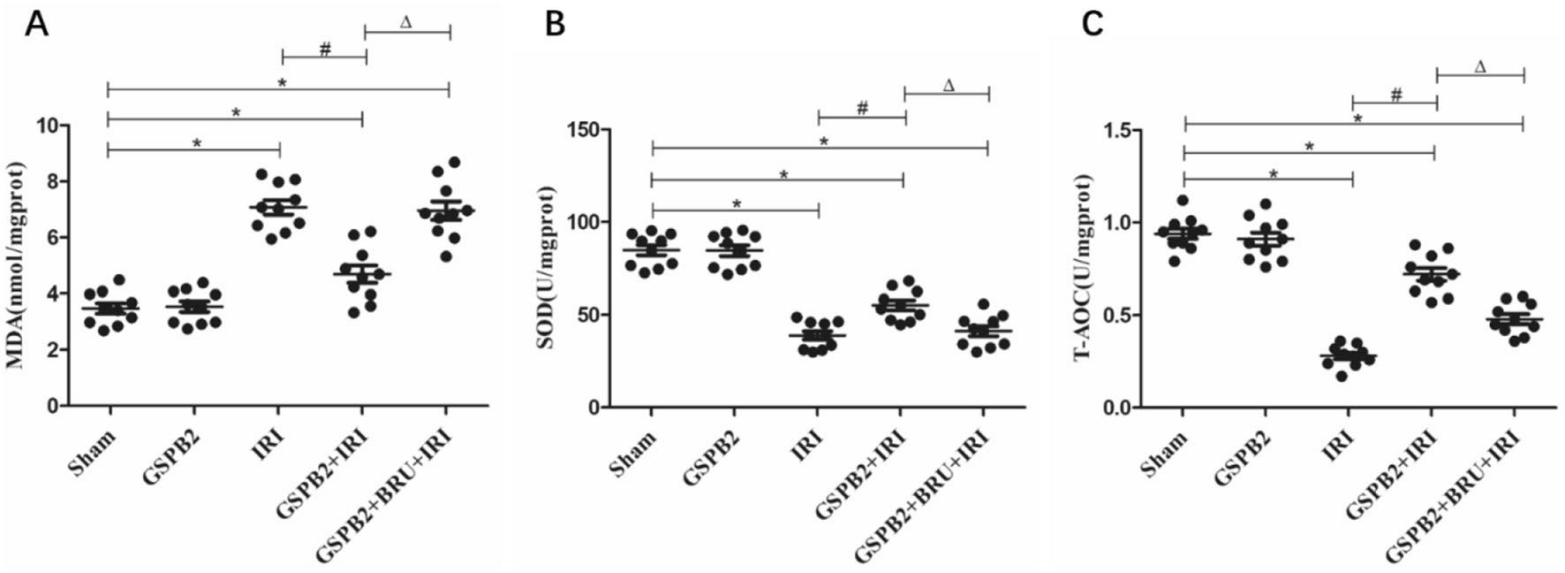
The levels of MDA, SOD and T-AOC in kidney of each group. **a** The level of MDA. **b** The level of SOD. **c** The level of T-AOC. (*n* = 10 in each group). *P < 0.05 versus group Sham, ^#^*P* < 0.05 versus group IRI. Δ*P* < 0.05 versus group GSPB2 + IRI. *SOD* superoxide dismutase, *MDA* malondialdehyde, *T-AOC* total antioxidant capacity

Compared to sham group, the levels of MDA in the kidney tissue of mice in IRI group, GSPB2 + IRI group and GSPB2 + BRU + IRI group were significantly increased, and the differences were statistically significant (*P* < 0.05). Meanwhile, compared to sham group, the levels of SOD and T-AOC in the kidney tissue of mice in IRI group, GSPB2 + IRI and GSPB2 + BRU + IRI group were significantly reduced. Furthermore, compared to IRI group, the levels of SOD and T-AOC protein in the kidney tissue of GSPB2 + IRI group were significantly increased, and the differences were statistically significant (*P* < 0.05). At the same time, compared to GSPB2 + IRI group, the levels of SOD and T-AOC in the kidney tissue of GSPB2 + BRU + IRI group were significantly reduced, and the difference were statistically significant (*P* < 0.05). See Fig. 5.

### Grape seed proanthocyanidin B2 pretreatment could significantly enhance the expression of antioxidant protein after renal ischemia–reperfusion injury

**Fig. 6:**
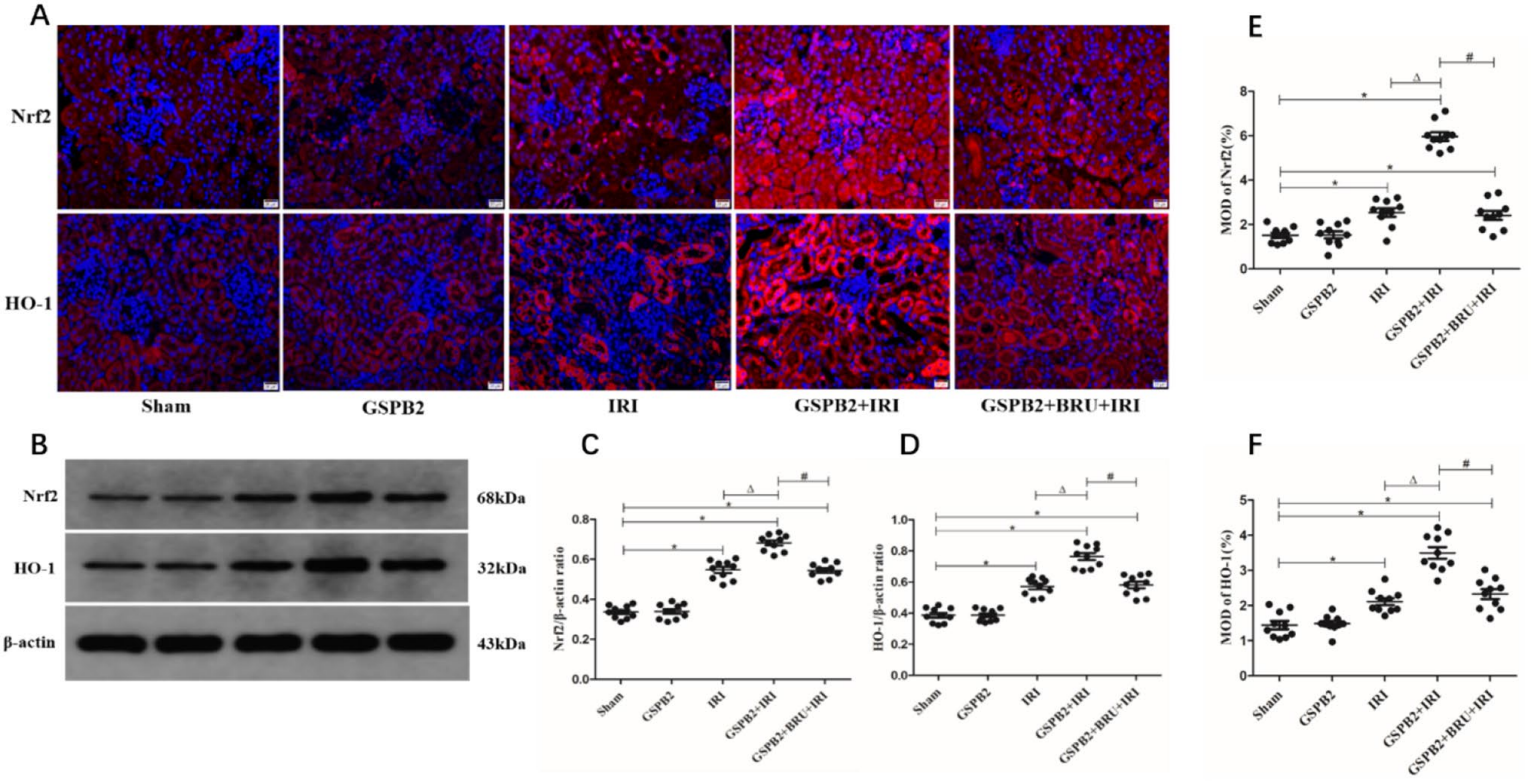
Grape seed proanthocyanidin B2 pretreatment could significantly increase the protein expression levels of Nrf2 and HO-1 in renal tissue of cortical region after renal ischemia–reperfusion according to the representative micrographs of immunofluorescence staining (× 400) and western-blotting. **A**, **E**, **F** Representative micrographs of immunofluorescence staining and quantitative analysis of Nrf2 and HO-1. **B–D** The expression levels of Nrf2 and HO-1 as assessed by western blotting. (*n* = 10 in each group). *P < 0.05 vs. group Sham. Δ*P* < 0.05 vs. group IRI. ^#^*P* < 0.05 vs. group GSPB2 + IRI

The results of immunofluorescence staining and western-blotting experiments in this study showed that compared with IRI group, the expression levels of Nrf2 and HO-1 protein in the kidney tissue of GSPB2 + IRI group mice were significantly up-regulated, and the difference was statistically significant (*P* < 0.05). At the same time, compared with GSPB2 + IRI group, the expression levels of Nrf2 and HO-1 protein in the kidney tissue of GSPB2 + BRU + IRI group were significantly down-regulated, and the difference was statistically significant (*P* < 0.05). As shown in Fig. 6.

### Grape seed proanthocyanidin B2 pretreatment could significantly inhibit the expression of endoplasmic reticulum stress-related proteins after renal ischemia–reperfusion injury

**Fig. 7:**
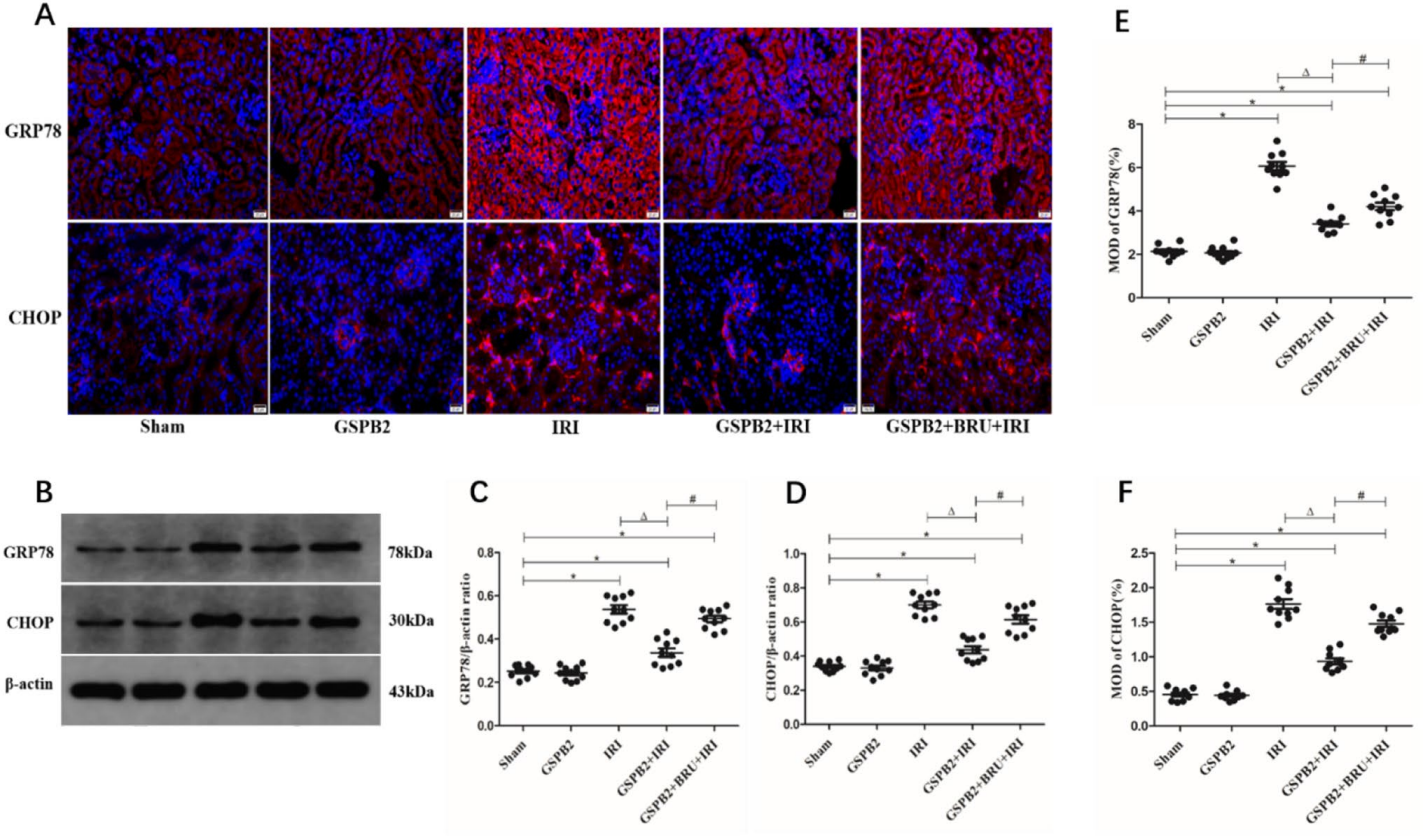
Grape seed proanthocyanidin B2 pretreatment could significantly restrain the protein expression levels of GRP78 and CHOP in renal tissue of cortical region after renal ischemia–reperfusion according to the representative micrographs of immunofluorescence staining (× 400) and western-blotting. **A**, **E**, **F** Representative micrographs of immunofluorescence staining and quantitative analysis of GRP78 and CHOP. **B–D** The expression levels of GRP78 and CHOP as assessed by western blotting. (*n* = 10 in each group). *P < 0.05 vs. group Sham. Δ*P* < 0.05 vs. group IRI. ^#^*P* < 0.05 vs. group GSPB2 + IRI

Compared to sham group, the expression levels of GRP78 and CHOP protein in the kidney tissue of mice in IRI group, GSPB2 + IRI group and GSPB2 + BRU + IRI group were significantly increased, and the difference was statistically significant (*P* < 0.05). Compared to IRI group, the expression levels of GRP78 and CHOP protein in the kidney tissue of GSPB2 + IRI group were significantly reduced, and the difference was statistically significant (*P* < 0.05). At the same time, compared with GSPB2 + IRI group, the GRP78 and CHOP protein expression levels in the kidney tissue of GSPB2 + BRU + IRI group were significantly increased, and the difference was statistically significant (*P* < 0.05). As shown in Fig. 7.

## Discussion

The pathophysiology and pathogenesis of ischemia–reperfusion injury involve multiple factors and are complex. The pathological process of ischemia–reperfusion injury involves ischemia, hypoxia, innate immune response, inflammation, apoptosis, necrosis, autophagy, oxidative stress injury, mitochondrial dysfunction, endoplasmic reticulum stress, etc., and also includes multiple participation of various signaling pathways [25–27]. In this process, oxidative stress damage, mitochondrial damage and endoplasmic reticulum stress play an important role. It is currently believed that a large amount of reactive oxygen species (ROS) can be produced during the reperfusion phase, and mitochondria are increasingly considered to be the key source of these ROS. These findings have been confirmed in many tissue types, including the kidney [28, 29]. Excessive superoxide produced by mitochondria can activate a variety of pathways and cause tissue damage. Among them, lipid peroxidation can cause cell or mitochondrial damage and further lead to interruption of adenosine triphosphate (ATP) production, imbalance of calcium ion levels, opening of mitochondrial permeability transition pores and subsequent apoptosis or necrosis [30]. Therefore, reducing the production of mitochondrial ROS or reducing oxidative stress damage and reducing the necrosis and apoptosis of renal tubular epithelial cells are of great significance to alleviating the ischemia–reperfusion injury of kidney tissue.

Under normal circumstances, mitochondrial respiration of renal tubular epithelial cells can produce ROS. Low levels of ROS, as an important intracellular and intercellular signal, are of great significance for maintaining the stability and function of the kidney's internal environment. These functions include maintaining renal hemodynamics, glomerular filtration rate, renal tubular reabsorption, and hormone secretion, etc., while excessive ROS can cause oxidative stress damage and harm the kidney tissue [31]. Studies have shown that procyanidin B2 has a strong antioxidant effect, and has good bioavailability in the body. Procyanidin B2 treatment can significantly promote the elimination of excessive ROS in cells, and can significantly increase the level of SOD in serum and intestinal tissues of mice, and reduce the level of MDA [22]. Liu JX’s research has shown that proanthocyanidin B2 intervention can promote Nrf2 nuclear translocation and have a protective effect on sepsis-related acute kidney injury mitochondrial dynamics [32]. At the same time, procyanidin B2 intervention can significantly reduce superoxide (O^2−^) And NOX4 levels, increase the levels of antioxidant enzymes SOD and GSH in kidney tissues, while reducing the level of MDA [32]. In this study, compared with the IRI group, the mitochondrial damage of renal tubular epithelial cells in the GSPB2 + IRI group was significantly alleviated. At the same time, the levels of antioxidant enzyme SOD and T-AOC in the mouse renal tissue were significantly increased. Meanwhile, the level of MDA in renal tissue was significantly reduced. This is similar to our previous research results of grape seed proanthocyanidin B2 intervention in renal ischemia–reperfusion injury of mice model [33].

In order to maintain cell redox homeostasis and avoid harmful oxidative stress injury, the endogenous antioxidant system composed of Kelch-like ECH related protein 1 (Keap1) and nuclear factor E2 related factor 2 (Nrf2) plays an important protective role by regulating the expression of a variety of detoxification related genes. Among them, Nrf2 is the main transcriptional regulator regulating redox state and antioxidant effect [34, 35]. Keap1 is a subunit protein of Nrf2 specific E3 ubiquitin ligase. Keap1 molecule contains specific cysteine residues, which can sense cellular oxidative stress by adding oxidants and electrophiles. Under normal circumstances, Keap1 protein and Nrf2 protein bind in the cytoplasm, and Nrf2 is regulated by Keap1 and undergoes ubiquitination and degradation to maintain a low level of Nrf2. When stimulated by oxidative stress, electrophilic reagent and cysteine residue of Keap1 are added to induce the change of spatial conformation of Keap1, so as to dissociate Nrf2 from Keap1, transfer the dissociated Nrf2 to the nucleus, combine with the antioxidant reaction elements in the nucleus, and enhance the expressions of heme oxygenase 1 (HO-1), NADPH quinone oxidoreductase 1 (NQO-1) and glutamate cysteine ligalytic subunit (GCLC), glutamate cysteine ligase modifier subunit (GCLM) and other antioxidant genes [36]. In this study, compared with the IRI group, the expression levels of Nrf2 and HO-1 protein in the ischemia–reperfusion injury kidney tissue of mice in the GSPB2 + IRI group were significantly increased. When Nrf2 inhibitor brusatol was added, the expression levels of Nrf2 and HO-1 protein in the ischemia–reperfusion injury kidney tissue of mice in the GSPB2 + BRU + IRI group were significantly lower than those in the GSPB2 + IRI group. The results of this study show that grape seed proanthocyanidin B2 pretreatment can activate the Nrf2/HO-1 signaling pathway in the antioxidant system and increase the expression levels of antioxidant proteins Nrf2 and HO-1, and help to reduce the renal ischemia–reperfusion-induced pathological or morphological damage and oxidative stress damage of tubular epithelial cells. In fact, systemic Nrf2 knockout mice have been used in a variety of disease models to study the role of Nrf2 in the kidney, including ischemia–reperfusion injury models [37], unilateral ureteral obstruction [38], and diabetic nephropathy [39], podocyte damage [40], autoimmune nephritis model, etc. [41]. The results of these models have shown that the loss of Nrf2 gene will aggravate the damage of kidney tissue or cells.

Endoplasmic reticulum stress is inevitable in the process of acute kidney injury induced by ischemia–reperfusion. In fact, under normal physiological conditions, the endoplasmic reticulum, as an important organelle, participates in the folding, assembly, modification, protein secretion, lipid synthesis, and calcium storage of cells. Studies have shown that when endothelial cells or epidermal cells are stimulated by stress, the unfolded protein or misfolded protein accumulated in the endoplasmic reticulum will trigger the unfolded protein response [42, 43]. The unfolded protein response is an adaptive protective mechanism that restores tissue or cell homeostasis by activating signal proteins such as PERK, IRE1, and ATF6 [43]. The unfolded protein response can inhibit overall protein translation through phosphorylation of eIF2α, increase the folding ability of endoplasmic reticulum protein by upregulating the endoplasmic reticulum chaperones, and increase the degradation of the endoplasmic reticulum, thereby providing short-term acute stress protection for tissues or cells [44]. When the endoplasmic reticulum is stimulated by severe stress such as excessive ROS and or Ca^2+^ overload, the homeostasis of the endoplasmic reticulum is impaired, resulting in the accumulation of unfolded and misfolded proteins. These changes can eventually lead to endoplasmic reticulum dysfunction and apoptosis [45]. GRP78 is a binding immunoglobulin and is often used as one of endoplasmic reticulum stress markers. Under normal physiological conditions, GRP78 and PERK, ATF-6, and IRE1 proteins bind to the endoplasmic reticulum [46]. When the homeostasis of the endoplasmic reticulum is disrupted, it can cause the accumulation of unfolded proteins in the endoplasmic reticulum, leading to the dissociation of GRP78 from PERK, ATF-6, and IRE1 proteins, and activating the endoplasmic reticulum stress signal transduction pathway [47]. CHOP is a transcription factor belonging to the C/EBP family. Under normal physiological conditions, CHOP is in a low expression state. The transcription and translation of CHOP are mainly regulated by IRE1α, ATF-6 and PERK [48, 49]. Studies have shown that CHOP plays an important role in cell apoptosis caused by endoplasmic reticulum stress induced by ischemia–reperfusion [50]. Studies have shown that intermedin intervention can reduce the apoptosis of renal tubular epithelial cells caused by hypoxia and reoxygenation by inhibiting the expression of GRP78, CHOP and caspase-12, and can significantly improve the renal function of rats after renal ischemia–reperfusion injury [51]. Nie X et al. found that procyanidin B2 pretreatment could activate PPAR δ- AMPK signaling pathway and significantly reduce the endoplasmic reticulum stress injury of human umbilical vein endothelial cells induced by high glucose or tunicamycin by inhibiting the expression of CHOP, GRP78, ATF4 and ATF3 genes [52]. In this study, grape seed procyanidin B2 pretreatment could significantly inhibit the expression of GRP78 and CHOP protein in renal tissue after ischemia–reperfusion injury and reduce the stress injury of endoplasmic reticulum induced by ischemia–reperfusion. At the same time, it could significantly inhibit the expression of apoptosis related protein cleaved-caspase3, obviously reduce the apoptosis of renal tubular epithelial cells induced by ischemia–reperfusion, and significantly improve the renal function after renal ischemia–reperfusion injury in mice.

## Conclusion

Grape seed proanthocyanidin B2 pretreatment can significantly reduce the oxidative stress injury and endoplasmic reticulum stress of mouse renal tubular epithelial cells, reduce the apoptosis of renal tubular epithelial cells, and significantly improve the renal function after renal ischemia–reperfusion injury. This may be related to the fact that grape seed proanthocyanidin B2 pretreatment can activate Nrf2 / HO-1 signal pathway in the antioxidant system of renal tubular epithelial cells, inhibit the expression of endoplasmic reticulum stress-related proteins GRP78, CHOP and apoptosis protein cleaved caspase3. However, the results of this experiment still need to be further verified by more mammalian experiments.

## Data Availability

Data sharing is not applicable to this article as no datasets were generated or analyzed during the current study.
